# Picture of thyroid cancer in 2012

**DOI:** 10.1186/1756-6614-6-S2-A29

**Published:** 2013-04-05

**Authors:** Aldona Kowalska

**Affiliations:** 1 Holycross Cancer Centre in Kielce, Poland

## 

Thyroid cancer (TC) is the most common tumour of the endocrine system. In Poland, the standardized incidence rate in 2010 was 6.7 for women, 1.5 for men and it is characterized by a constant increase. Patients with thyroid cancer make up a very heterogeneous group with a differential clinical course - from an indolent to a fast progressing which is the cause of a patient’s death.

As our studies indicate, in recent years papillary thyroid cancer with a diameter of less than 10 mm dominates among the cases of differentiated thyroid carcinoma (DTC). Such situation requires the verification of the approach to the diagnostic process - moving the diagnostic burden from a palpable examination to an ultrasound method and indicating an outbreak to cytological and molecular examination. The open problem is reducing the diameter of lesions displayed in ultrasonography, which should be subjected to further assessment.

Surgical treatment with a total thyroidectomy with central lymph node dissection is a common procedure. Supplementary treatment with ^131^I has a documented value in the cases of advanced TC. Controversy arouses with the usage of ^131^I in patients with a lower stadium. The results of ESTIMABL and HiLo studies indicate high effectiveness of ablation with the use of an isotope with an activity of 30mCi, regardless of endogenous or exogenous TSH stimulation.

While monitoring the patients with DTC, the main tool is an ultrasound examination of a neck and thyroglobulin (TG) measurement. High hopes are connected with the use of ultrasensitive TG measurements under exogenous or endogenous TSH stimulation.

Isotope imaging is reserved only for patients with the suspicion of a relapse or metastases. To examinations used in these cases, a PET technique with the use of ^124^I or FDG was joined.

Due to a very differentiated clinical course of TC, prognostic indicators of worse prognoses requiring more aggressive measures are searched for. High expectations were connected with a BRAF mutation. Many studies have indicated that the BRAF^V600E^ mutation can correlate with a more aggressive clinical course of papillary thyroid cancer and with a worse prognosis. The results of our studies seem not to confirm this opinion - the high mutation frequency (72.5%) in papillary thyroid cancer of very good prognosis with a diameter of less than 10 mm.

Recent years have brought new therapeutic possibilities for the patients with the spread of the disease and with the lack of iodine uptake. A new group of medications are tyrosine kinase inhibitors out of which Vandetanib owns a registration to treat patients with medullary thyroid cancer. The other agents are in the course of clinical studies.

The most important problems for the future are: in the field of diagnostics - the development of molecular methods which improve the accuracy of cytological diagnosis; in the field of therapy - the identification of prognostic indicators of a worse disease course and a more aggressive treatment of this group of patients; seeking new medications aiming according to the pathway of oncogenesis.

